# Persistently Elevated Right Ventricular Index of Myocardial Performance in Preterm Infants with Incipient Bronchopulmonary Dysplasia

**DOI:** 10.1371/journal.pone.0038352

**Published:** 2012-06-01

**Authors:** Christoph Czernik, Stefanie Rhode, Boris Metze, Gerd Schmalisch, Christoph Bührer

**Affiliations:** Department of Neonatology, Charité University Medical Center, Berlin, Germany; University of Giessen Lung Center, Germany

## Abstract

**Objectives:**

Elevated pulmonary vascular resistance occurs during the first days after birth in all newborn infants and persists in infants at risk for bronchopulmonary dysplasia (BPD).

It is difficult to measure in a non-invasive fashion. We assessed the usefulness of the right ventricular index of myocardial performance (RIMP) to estimate pulmonary vascular resistance in very low birth weight infants.

**Study Design:**

Prospective echocardiography on day of life (DOL) 2, 7, 14, and 28 in 121 preterm infants (median [quartiles] gestational age 28 [26]–[29] weeks, birth weight 998 [743–1225] g) of whom 36 developed BPD (oxygen supplementation at 36 postmenstrual weeks).

**Results:**

RIMP derived by conventional pulsed Doppler technique was unrelated to heart rate or mean blood pressure. RIMP on DOL 2 was similar in infants who subsequently did (0.39 [0.33–0.55]) and did not develop BPD (0.39 [0.28–0.51], p = 0.467). RIMP declined steadily in non-BPD infants but not in BPD infants (DOL 7: 0.31[0.22–0.39] vs. 0.35[0.29–0.48], p = 0.014; DOL 14: 0.23[0.17–0.30] vs. 0.35[0.25–0.43], p<0.001; DOL 28: 0.21[0.15–0.28] vs. 0.31 [0.21–0.35], p = 0.015).

**Conclusions:**

In preterm infants, a decline in RIMP after birth was not observed in those with incipient BPD. The pattern of RIMP measured in preterm infants is commensurate with that of pulmonary vascular resistance.

## Introduction

Bronchopulmonary dysplasia (BPD) remains the most common long-term complication of very preterm birth [1]. In contrast to the type of BPD observed in the pre-surfactant era, featuring airway inflammation, fibrosis, and smooth muscle hypertrophy, the hallmark of the current “New BPD” is delayed alveolarization with decreased numbers of fully developed alveoli and alveolar capillary hypoplasia [2]–[4]. Premature infants at greatest risk for BPD are born before 28 weeks, during the late canalicular or saccular stage of lung development, just as the airways become juxtaposed to the pulmonary vessels. Various animal models of BPD and autopsy studies of humans who died from BPD have consistently shown a reduction in the number of small arteries and an abnormal distribution of vessels within the distal lung [5]–[7]. In addition to dysmorphic growth, the pulmonary vasculature in BPD undergoes hypertensive structural remodeling, which includes medial hypertrophy and distal muscularization of small peripheral arteries [8]. Abnormal vasoreactivity, reduced arterial number, and structural abnormalities of the vessel wall can contribute to pulmonary hypertension in BPD, leading to significant morbidity and mortality [9]. With time, dysfunction of the right ventricle (RV) or outright cor pulmonale may develop. Early assessment of RV function would greatly assist in the supportive care of these infants but the echocardiographic parameters routinely used lack the level of sensitivity needed to reliably indicate increased pulmonary vascular resistance. Tricuspid regurgitation to estimate RV pressure is accurate in identifying patients with chronic pulmonary hypertension in only 30% to 73% of cases [10], [11].

An easily measured Doppler index (a ventricular index of myocardial performance, sometimes called the Tei index [12]) that would combine systolic and diastolic time intervals was recently proposed. This index is applicable to both the right and left ventricles. It is defined as the sum of isovolumetric contraction time and isovolumetric relaxation time divided by ejection time. Several authors have reported the usefulness of the right ventricular index of myocardial performance (RIMP) for assessing RV function in adults [13], [14], whereas only a few studies have assessed RIMP in neonates and children [15]–[18]. Moreover, it has been well established that RIMP correlates with invasively measured pulmonary vascular resistance (PVR) in adults [19]–[21], but that the invasive measurement of PVR in neonates is difficult to perform.

Pulmonary vascular resistance is high at birth and then declines during the first weeks of life in infants without major pulmonary morbidity. In contrast, incipient or established BPD is characterized by elevated pulmonary resistance. We hypothesized that this pattern is reflected in RIMP values serially obtained in very low birth weight (VLBW) infants with BPD compared to infants without BPD.

## Patients and Methods

### Patients

**Figure 1:**
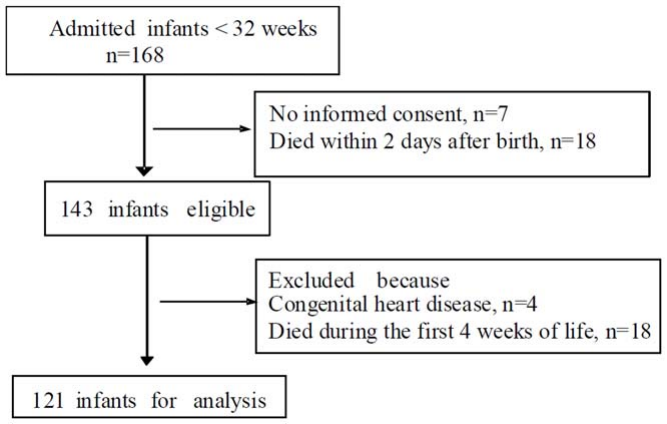
Flow diagram of the study population.

A prospective study was conducted at a single tertiary care perinatal center from September 2008 to January 2010. For this study we enrolled 121 of the 168 admitted preterm infants born at our hospital and below <32 weeks gestational age and birth weight <1500 g (Figure 1). 36 infants had been diagnosed with BPD based on their requirement for supplemental oxygen to maintain preductal arterial oxygen saturations of 92% at 36 weeks postmenstrual age [22], [23].

Infants who had a congenital heart malformation or infants who died within 48 h of birth or infants who died within the first month of life and therefore did not undergo the full set of four sequential echocardiograms were excluded.

All infants received standard intensive care and no infant required sedation or muscle relaxation for mechanical ventilation. The study protocol was approved by the local institutional review board (Ethikkommission der Charité, # EA2/072/08), and written informed parental consent was obtained.

### Echocardiographic measurements

All patients were examined using a 3.9 to 6.9 MHz transducer interfaced with a Vingmed System Vivid 7 Dimension'06 (GE Vingmed, Horten, Norway). Transthoracic imaging was performed on the neonates at ages 2, 7, 14 and 28 days in a supine position without sedation.

**Figure 2:**
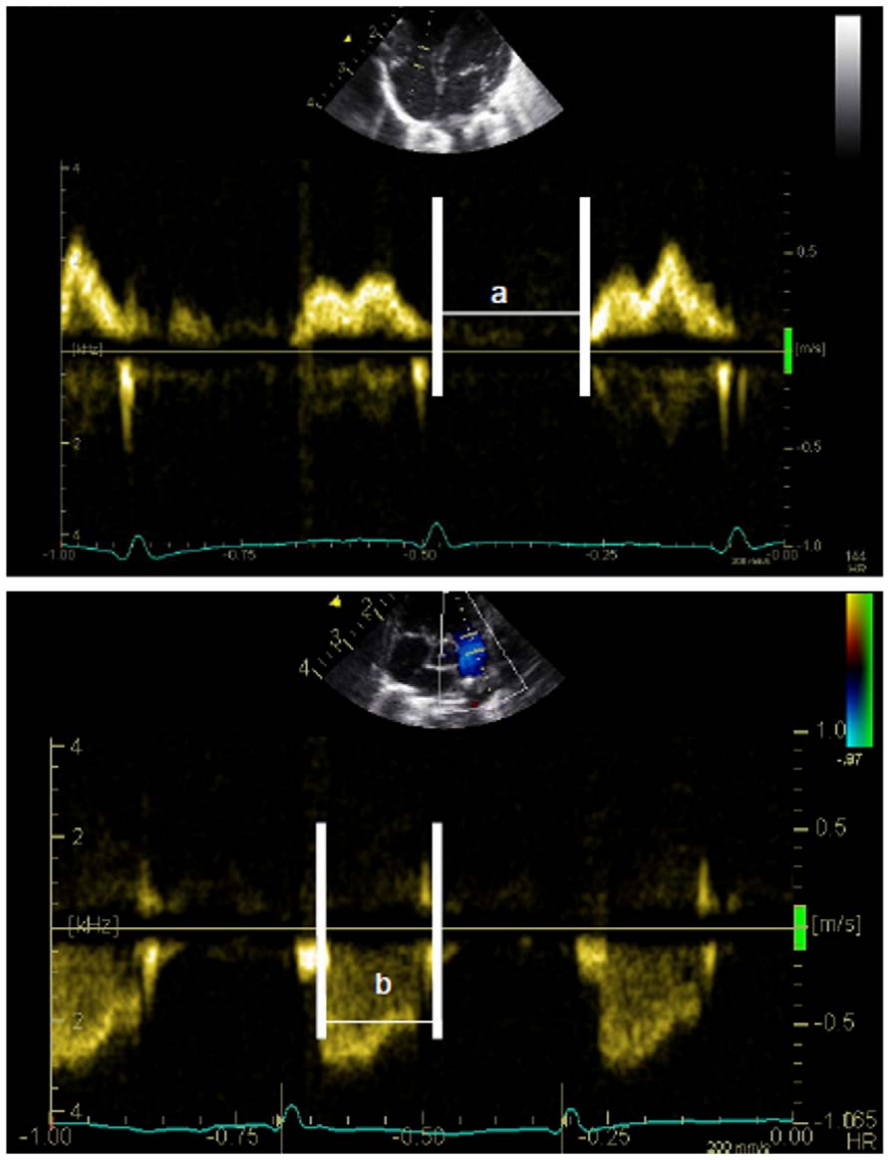
Doppler methodology. Measurement of the myocardial performance index by pulsed Doppler in the right ventricle capturing inflow and outflow. The “a” interval is the time from tricuspidal closing to opening, and the “b” interval is the ejection time in milliseconds.

The presence or absence of a patent ductus arteriosus (PDA) was determined by direct ductal imaging in the high left parasternal view with colour Doppler mapping. Doppler measurements of the RIMP were performed using the method proposed by Tei et al [12]. Pulsed-Doppler waveforms of tricuspid inflow were recorded from the parasternal four-chamber view, while the right ventricular outflow patterns by pulsed-Doppler were visualized from the parasternal short-axis view in accordance with the recommendations of the American Society of Echocardiography [24]. RIMP was calculated as (a–b)/b (Figure 2).

### Statistical analysis

Patient characteristics were described by rates (%) or median values and quartiles and compared using Fisher's exact test or the Mann-Whitney U-Test as appropriate. The echocardiographic parameters a and b were measured of three consecutive cardiac cycles. The calculated means were used to determine RIMP and the coefficient of variation was used to describe the intra-observer variability presented by median and quartiles. Offline analysis of the data was performed using a special software program (EchoPac Vingmed, Horten, Norway). All measurements were performed by a single, non-blinded investigator (S.R.), precluding any inter-observer variability. The RIMP of the patient groups was described by median and quartiles and compared using the Mann-Whitney U-Test. Spearman rank order correlation coefficients were calculated to investigate the impact of clinical and hemodynamic parameters on RIMP at the 4 measurement time points. The Friedman Test was used to investigate the age dependency of RIMP. Statistical analysis was performed using SPSS (Version 18, SPSS Inc. Chicago, IL, USA). A p value of <0.05 was considered statistically significant.

## Results

### Patient characteristics

Table 1 compares neonatal patient characteristics between infants with and without BPD. BPD infants had a significantly lower gestational age and birth weight (p<0.001). Males were more likely to develop BPD than females (p = 0.046). The rate of fetal lung maturation by prenatal steroid administration (considered complete if the mother had received two injections of 12 mg betamethasone 24 hours apart and more than 24 hours before delivery) did not differ significantly between the patient groups. Preterm infants with BPD received significantly longer mechanical ventilation (p<0.001), supplemental oxygen (p<0.001), and hospitalization and had a higher mortality rate (p = 0.029). Three infants died after 36 weeks gestational age but prior to discharge.

**Table 1:** Patient characteristics (presented as median values with quartiles (in brackets) or N (%)).

	Non-BPD infants	BPD infants	P
	N = 85	N = 36	
Gestational age (weeks)	29(27–30)	26 (24–27)	**<0.001**
Birth weight (g)	1100 (890–1300)	667 (585–871)	**<0.001**
Male	36 (42%)	23 (64%)	**0.046**
Prenatal steroids	80 (94%)	34(94%)	0.944
Surfactant administration	71(84%)	35(97%)	**0.038**
Mechanical ventilation (days)	3 (1–5)	31 (10–37)	**<0.001**
Oxygen treatment (days)	4 (1–12)	89 (52–104)	**<0.001**
Hospitalization (days)	58 (45–75)	93 (78–108)	**<0.001**
Alive at discharge	85 (100%)	33 (92%)	**0.029**

Statistically significant p-values are printed in bold.

### Reliability of RIMP measurements

We were unable to assess the RIMP successfully by Doppler at all measurement time points because of an unclear waveform depicting the right ventricular inflow or outflow. The assessment success rates at day of life (DOL) 2, 7, 14 and 28 were 78%, 73%, 67% and 79%, but there were no statistically significant differences in the drop out frequency between the patient groups at the four time points. The intra-observer variability of the both measurement parameters a and b was comparable with a median (quartiles) coefficient of variation of 3.4% (2.0%–5.1%) for a and 4.2% (2.7%–6.4%) for b.

### Influencing factors on RIMP

RIMP was strongly age dependent (p<0.001) and decreased continuously during the neonatal period. Table 2 shows the correlation of RIMP with clinical and hemodynamic parameters at the four time points of measurement in our study population. RIMP did not correlate with gestational age and birth weight at day 2, but the correlation with statistical significance increases with increasing age. However, the correlation of RIMP with gestational age and birth weight disappeared if we analysed infants with and without BPD separately. The RIMP was independent of heart rate and mean blood pressure at all measurement time points.

**Table 2:** Correlation of RIMP with clinical and hemodynamic parameters at the 4 time points of measurement (Spearman rank order correlation coefficients and p values).

	RIMP	RIMP	RIMP	RIMP
	DOL 2	DOL 7	DOL 14	DOL 28
Gestational age (weeks)	0.018	**−0.214**	**−0.441**	**−0.229**
	P = 0.860	**P = 0.046**	**P<0.001**	**P = 0.026**
Birth weight (g)	0.070	**−0.249**	**−0.4446**	**−0.257**
	P = 0.501	**P = 0.020**	**P<0.001**	**P = 0.013**
Heart rate (bpm)	−0.098	−0.016	0.189	−0.086
	P = 0.344	P = 0.880	P = 0.090	P = 0.415
Mean blood pressure (mmHg)	−0.035	−0.045	−0.193	−0.129
	P = 0.739	P = 0.685	P = 0.090	P = 0.233

Statistically significant p values are printed in bold.

### RIMP and BPD

**Figure 3:**
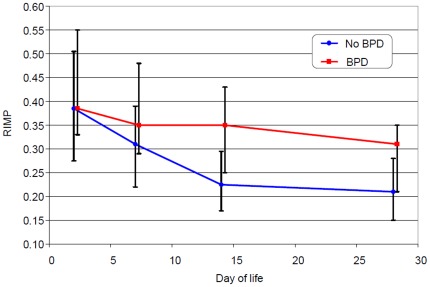
Decrease in RIMP during the neonatal period in infants who did or did not develop BPD (median and quartile values presented).

The decrease in RIMP during the first two weeks of life differed significantly between non-BPD infants and those infants who developed a BPD as shown in Figure 3. RIMP values decreased faster in infants without BPD than in infants who developed BPD. In infants who did not develop BPD, a rapid decrease occurred during the first 2 measurement time points, whereas the RIMP of infants who developed BPD remained high. Between days 14 and 28 the decline of RIMP was approximately equal in both groups. At DOL 2 both groups exhibited similarly high RIMP values without statistical significance (0.39 [0.33–0.55] vs. 0.39 [0.28–0.51], p = 0.467). Over time RIMP remained higher in infants who developed BPD compared to infants without BPD (DOL 7: 0.31[0.22–0.39] vs. 0.35[0.29–0.48], p = 0.014; DOL 14: 0.23[0.17–0.30] vs. 0.35[0.25–0.43], p<0.001; DOL 28: 0.21[0.15–0.28] vs. 0.31 [0.21–0.35], p = 0.015).

### PDA and mechanical ventilation

The course of RIMP was age-dependent and differed between infants with and without BPD. Therefore the effect of PDA and mechanical ventilation was investigated in the subgroups with or without BPD. At DOL 2 all BPD infants had a PDA, so its impact on RIMP could not be investigated. In both patient groups defined by the presence or absence of BPD, there was no statistically significant effect of PDA on RIMP at DOL 7, 14, and 28 (Table 3). At DOL 28 no infant without BPD was mechanically ventilated, thus the effect of mechanical ventilation on RIMP could only investigated at DOL 2,7, and 14. At these time points of measurement in both patient groups there was a no statistically significant effect of mechanical ventilation on RIMP (Table 4).

**Table 3:** Effect of echocardiographically detectable PDA on RIMP at DOL 7, 14 and 28.

		DOL 7	DOL 14	DOL 28
*BPD infants*	Without PDA	0.32 (0.23–0.49)	0.26 (0.25–0.35)	0.29 (0.21–0.35)
	With PDA	0.35 (0.3–0.47)	0.38 (0.24–0.44)	0.31 (0.16–0.35)
	P-value	0.315	0.275	0.765
*Non-BPD infants*	Without PDA	0.31 (0.24–0.38)	0.23 (0.18–0.29)	0.21 (0.15–0.26)
	With PDA	0.31 (0.21–0.41)	0.22 (0.13–0.30)	0.22 (0.15–0.38)
	P-value	0.687	0.543	0.512

**Table 4:** Effect of mechanical ventilation on RIMP at day DOL 2, 7 and 14.

		DOL 2	DOL 7	DOL 14
*BPD infants*	Without mechanical ventilation	0.43 (0.38–0.48)	0.33 (0.25–0.49)	0.28 (0.26–0.40)
	With mechanical ventilation	0.38 (0.33–0.57)	0.39 (0.30–0.48)	0.37 (0.21–0.44)
	P-value	0.670	0.340	0.620
*Non-BPD infants*	Without mechanical ventilation	0.39 (0.29–0.50)	0.31 (0.21–0.39)	0.21 (0.17–0.29)
	With mechanical ventilation	0.40 (0.27–0.51)	0.31 (0.28–0.41)	0.25 (0.23–0.30)
	P-value	0.917	0.577	0.362

## Discussion

Our data confirm the hypothesis that RIMP differs between VLBW infants without BPD and infants who develop BPD between 7 and 28 days of life. RIMP values were significantly higher on DOL 7, 14 and 28 in infants with a subsequent diagnosis of BPD compared to controls.

Our results reveal that average RIMP values in VLBW infants are relatively high at DOL 2 but fall rapidly during the neonatal period in preterm VLBW infants similar to the drop observed in term and near-term infants and confirmed the longitudinal time course of RIMP during the neonatal period as already demonstrated by Murase et al. [18], however, in the present study the RIMP values were slightly higher. We assume that the elevated RIMP values in the cohort presented here is linked to the high proportion of BPD infants while the percentage of BPD was not specified by Murase et al. [18]. Sample size appears to matter as well, as Yates et al. [11] found no marked difference in RIMP between infants with (n = 15) and without BPD (n = 6) using the tissue Doppler imaging technique.

These time course changes in RIMP reflect a differential decline of pulmonary vascular resistance in infants with a BPD diagnosis. VLBW infants who developed a BPD showed only a slight decrease in RIMP during the first month of life compared to infants without BPD.

It is known that several cardiovascular changes take place during the early neonatal period that involve the adaptation of myocardial performance, such as the closure of fetal shunts, increased pulmonary circulation, and increased peripheral systemic circulatory resistance. VLBW infants in particular are at higher risk of hemodynamic instability in postnatal cardiovascular adaptation and for developing BPD [25]. We did not investigate the commonly used echocardiographic parameters (e.g. tricuspid regurgitant jet, septal displacement, or right atrial enlargement) because their sensitivity to assess right ventricular performance in preterm newborn infants is poor [10], [11] and much lower than in adults. Moreover, disappearance of tricuspid regurgitation and ductal closure in most infants by day 10 precludes these methods after the second week of life [26], [27].

Especially shortly after birth, PVR and pulmonary arterial pressure fall rapidly, whereas pulmonary blood flow reaches systemic levels. The exact mechanisms by which these dramatic changes in PVR occur with the onset of ventilation at birth have been investigated extensively [28]. These early changes in PVR are reflected by the decline in RIMP in our study. In contrast, the pulmonary vasculature in infants who develop BPD is characterized by reduced arterial number, medial hypertrophy and distal muscularization of small peripheral arteries, and abnormal vasoreactivity. These changes inhibit the decline of RIMP observed in infants without major pulmonary disease.

The observation that RIMP represents global RV function was already reported by Sugiura et al. from recent animal studies. Global RV function is always regulated by both myocardial performance and pulmonary artery pressure as RV afterloads [17].

Several authors reported that RIMP is readily available in tachycardia, independent of heart rate or blood pressure, and not affected by tricuspid regurgitation [15], [29]. Our study results confirmed these findings. We found no correlations between RIMP and heart rate or blood pressure. Thus measuring RIMP in neonates, who normally have high heart rates, is feasible.

In contrast, the most widely used echocardiographic parameter, the “E/A ratio”, for evaluating RV diastolic function in adults [12], [30], not widely used in children because it is difficult to obtain in tachycardia, is related to heart rate. The significant inverse correlation between RIMP and birth weight and gestational age after day 7 is apparently caused by the increased values of RIMP of BPD infants who have significant lower birth weights and gestational ages (Table 1), as this correlation disappears if patients are stratified according to BPD. When analyzing both patient groups separately, we found no statistically significant influence of PDA or mechanical ventilation on RIMP.

Despite the fact that current therapy during the neonatal period includes surfactant replacement, supportive ventilation strategies with lower mean airway pressures and permissive hypercapnia, inhaled and systemic corticosteroid therapy, bronchodilators, diuretic therapy, vitamin A therapy, and optimal nutrition, BPD remains a major cause of morbidity and mortality among VLBW infants. At the moment, there is no “magic” biomarker for the early identification of infants who subsequently develop BPD [31]. RIMP measurements could be helpful as a diagnostic tool for assessing RV function and in guiding the titration of drug therapy, because optimal diuretic dosages are also still unclear.

A limitation of our study is that the interval between the end and onset of tricuspid inflow and the ejection time are measured sequentially and not during the same cardiac cycle. Future investigations might compare RIMP obtained by conventional pulsed Doppler echocardiography with data derived from tissue Doppler echocardiography, which allows for the simultaneously measurement of both the diastolic and systolic intervals from the myocardium, or other methods to assess systolic and diastolic ventricular function. However, previous studies have already shown a relatively good correlation of RIMP obtained by pulsed Doppler and tissue Doppler echocardiography [32]. As BPD infants are more immature and receive more intensive care than non-BPD infants, and many of the medical interventions aimed to reduce preload and afterload may impact on RIMP.

In conclusion RIMP, which combines systolic and diastolic time intervals, is a feasible approach for assessing global RV function in VLBW infants. We have shown that RIMP values were significantly higher in infants with a subsequent diagnosis of BPD compared to controls indicating an increased pulmonary vascular resistance. Using RIMP as a sole marker of global RV function is currently not recommended by the American Society of Echocardiography [24]. However, continuing research to understand the limitations and advantages of RIMP measurement in infants with BPD may permit future incorporation of RIMP into clinical assessment of strategies to treat BPD.
